# Class V chitin synthase and β(1,3)-glucan synthase co-travel in the same vesicle in *Zymoseptoria tritici*

**DOI:** 10.1016/j.fgb.2019.103286

**Published:** 2020-02

**Authors:** Martin Schuster, Celia Guiu-Aragones, Gero Steinberg

**Affiliations:** a School of Biosciences, University of Exeter, Exeter EX4 4QD, UK; b 65 Vallcalent Street, 25006 Lleida, Spain

**Keywords:** Membrane trafficking, Chitin synthases, Glucan synthase, Cell wall formation

## Abstract

The fungal cell wall consists of proteins and polysaccharides, formed by the co-ordinated activity of enzymes, such as chitin or glucan synthases. These enzymes are delivered via secretory vesicles to the hyphal tip. In the ascomycete *Neurospora crassa*, chitin synthases and β(1,3)-glucan synthase are transported in different vesicles, whereas they co-travel along microtubules in the basidiomycete *Ustilago maydis.* This suggests fundamental differences in wall synthesis between taxa. Here, we visualize the class V chitin synthase ZtChs5 and the β(1,3)-glucan synthase ZtGcs1 in the ascomycete *Zymoseptoria tritici*. Live cell imaging demonstrate that both enzymes co-locate to the apical plasma membrane, but are not concentrated in the Spitzenkörper. Delivery involves co-transport along microtubules of the chitin and glucan synthase. Live cell imaging and electron microscopy suggest that both cell wall synthases locate in the same vesicle. Thus, microtubule-dependent co-delivery of cell wall synthases in the same vesicle is found in asco- and basidiomycetes.

## Highlights

• Native chitin (Chs5) and glucan synthase (Gsc1) visualised in the pathogen *Zymoseptoria tritici*.
• Chs5 and Gsc1 are transported along microtubules.
• Chs5 and Gsc1 do localise to the apical plasma membrane, but not the Spitzenkörper.
• Light and electron microscopy how co-travel of Chs5 and Gsc1 in the same secretory vesicle.
• Enzyme delivery in *Z. tritici* is different from *Neurospora crassa*, but similar to *Ustilago maydis*.

## 1 Introduction

Hyphal tip growth involves apical formation of the cell wall. This extracellular matrix is a complex meshwork of proteins and polysaccharides. The dominant sugar polymers are β(1,3)-glucan and chitin, synthesized by plasma membrane-located glucan synthases and chitin synthases, respectively (Geoghegan et al., 2017). Cell wall synthases are integral membrane-bound enzymes, which are delivered to the hyphal apex in vesicles, named chitosomes (Bartnicki-Garcia, 2006, Bracker et al., 1976, Sietsma et al., 1996). This transport involves relay of motor proteins along the fibers of the cytoskeleton (Steinberg, 2007). Upon arrival at the site of exocytosis, the enzymes are inserted into the plasma membrane via local vesicle fusion. Once secreted, the enzymes co-operate to form the complex cell wall.

Fungal genomes carry a large number of chitin synthases, which belong to different classes and have been shown to participate in various developmental stages (Fajardo-Somera et al., 2015, Kong et al., 2012, Weber et al., 2006). Pioneering live cell imaging studies by Riquelme and co-workers in *Neurospora crassa* demonstrated that fluorescently-labelled chitin synthases of different classes, accumulate in microvesicles (=chitosomes) at the core of the apical Spitzenkörper (SPK; (Fajardo-Somera et al., 2015, Riquelme et al., 2007, Sanchez-Leon et al., 2011), overview in (Steinberg et al., 2017)). The periphery of the SPK comprises larger macrovesicles, which contain β(1,3)-glucan synthase (Sanchez-Leon et al., 2015a, Sanchez-Leon et al., 2015b). Thus, both enzymes are delivered to the growing hyphal tip in different sub-populations of vesicles. The SPK may release these two populations of vesicles in a co-ordinated way to ensure that their cargo enzymes align in proximity for co-ordinated synthesis of the cell wall. However, studies in the corn smut fungus *Ustilago maydis* suggest an alternative concept. Here, live cell imaging and ultrastructural studies of native levels of class V chitin synthase and β(1,3)-glucan synthase revealed that both enzymes co-travel in the same ∼24 nm vesicle (Schuster et al., 2016). Fusion of such a vesicle with the plasma membrane closely aligns these enzymes. This supports cell wall synthesis, shown to begin immediately after exocytosis (Schuster et al., 2016). However, these results were obtained in yeast-like cells of *U. maydis*, and as such may not be directly comparable to the situation in hyphae of *N. crassa*. Moreover, *U. maydis* is a basidiomycete, whereas *N. crassa* is an ascomycete, which raises the possibility that delivery of cell wall synthases is mediated in different ways in the two taxa.

In this study, we visualize native levels of class V chitin synthase and β(1,3)-glucan synthase in living hyphae of the ascomycete *Zymoseptoria tritici*. We show that both enzymes move at similar rates along microtubules. Co-observation of green- and red-fluorescent fusion proteins, both in cells and purified vesicles, demonstrates that both enzymes are located in the same vesicle. Thus, co-delivery of cell wall synthases occurs in basidiomycete and ascomycete fungi.

## 2 Results and discussion

**Fig. 1:**
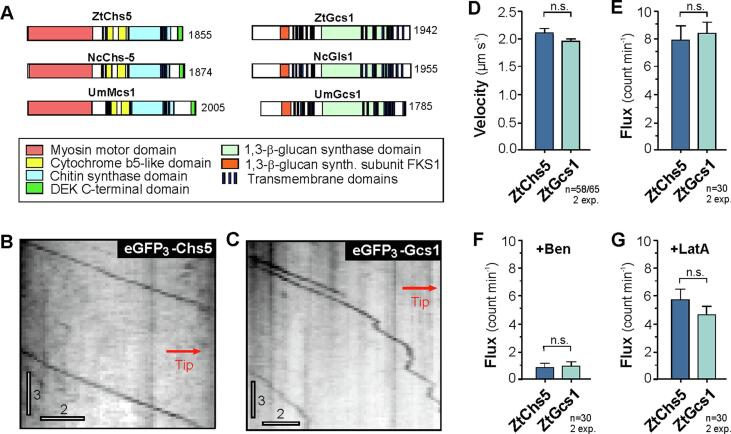
Motility of fluorescent chitin and glucan synthase in hyphae of *Z. tritici.* (A) Domain structure of class V chitin synthases and β(1,3)-glucan synthase in *Z. tritici* (ZtChs5, Genbank accession number: SMQ47822.1; ZtGcs1, Genbank accession number: SMR60639.1), *N. crassa* (NcChs-5, Genbank accession number: XP_956333.2; NcGls1, Genbank accession number: XP_957980.1) and *U. maydis* (UmMcs1, Genbank accession number: XP_011389642.1; UmGcs1, Genbank accession number: XP_011387626.1). (B) Contrast inverted kymograph showing directed motility of eGFP_3_-ZtChs5. The direction relative to the hyphal tip is indicated by red arrow. Horizontal bar: 2 µm, vertical bar: 3 s. (C) Contrast inverted kymograph showing directed motility of eGFP_4_-ZtGcs1. The direction relative to the hyphal tip is indicated by red arrow. Horizontal bar: 2 µm, vertical bar: 3 s. (D) Bar chart showing average velocities of moving eGFP_3_-ZtChs5 and eGFP_4_-ZtGcs1 signals in hyphae of *Z. tritici*. Bars represent mean ± SEM, sample size *n* is indicated. Shapiro-Wilk testing revealed non-normal data distribution (P ≤ 0.05) and comparison used a nonparametric Mann-Whitney test, with n.s. indicating non-significant difference (*P* = 0.1099). (E) Bar chart show the flux of eGFP_3_-ZtChs5 and eGFP_4_-ZtGcs1 to the hyphal tip. Bars represent mean ± SEM, sample size *n* is indicated. Shapiro-Wilk testing revealed non-normal data distribution (P ≤ 0.05) and comparison used a nonparametric Mann-Whitney test, with n.s. indicating non-significant difference (*P* = 0.0840). (F) Bar chart show the flux of eGFP_3_-ZtChs5 and eGFP_4_-ZtGcs1 to the hyphal tip in the presence of the MT inhibitor benomyl. Bars represent mean ± SEM, sample size *n* is indicated. Shapiro-Wilk testing revealed non-normal data distribution (P ≤ 0.05) and comparison used a nonparametric Mann-Whitney test, with n.s. indicating non-significant difference (*P* = 0.5798). (G) Bar chart show the flux of eGFP_3_-ZtChs5 and eGFP_4_-ZtGcs1 to the hyphal tip in the presence of the F-actin inhibitor latrunculin A. Bars represent mean ± SEM, sample size *n* is indicated. Shapiro-Wilk testing revealed non-normal data distribution (P ≤ 0.05) and comparison used a nonparametric Mann-Whitney test, with n.s. indicating non-significant difference (*P* = 0.3869). (For interpretation of the references to color in this figure legend, the reader is referred to the web version of this article.)

*Z. tritici* is the most important fungal wheat pathogen in temperate climate regions (Fones and Gurr, 2015), yet only very little is known about membrane trafficking and secretion in this fungus (Steinberg, 2015). We screened the previously published genome of *Z. tritici* and identified a putative class V chitin synthase (ZtChs5, for all Genbank accession numbers see legend of Fig. 1) and a putative β(1,3)-glucan synthase (ZtGcs1). ZtChs5 shares 64.7%/77.9% sequence identity/similarity with Chs-5 from *N. crassa* (Fajardo-Somera et al., 2015) and 38.2%/55.0% sequence identity with the class V chitin synthase from *U. maydis* (UmMcs1, Weber et al., 2006). The predicted β(1,3)-glucan synthase (ZtGcs1) shares 71.4%/81.6% sequence identity/similarity FKS-1 in *N. crassa* (Sanchez-Leon et al., 2015a, Sanchez-Leon et al., 2015b), and 53.9%/67.1% sequence identity/similarity UmGcs1 in *U. maydis* (Schuster et al., 2016). Thus, both *Z. tritici* proteins are much more closely related to the *N. crassa* than the *U. maydis* homologues, which reflects the evolutionary position of all three fungi, with *Z. tritici* and *N. crassa* belonging to the phylum Ascomycota and the subphylum Pezizomycotina, whereas *U. maydis* is a basidiomycete, belonging to the Ustilaginomycotina (Stajich et al., 2009). Despite these differences, all predicted proteins share a very similar domain organization (Fig. 1A).

We fused a triple tag of enhanced green fluorescent protein (eGFP_3_) to the endogenous *Ztchs5* and a quadruple enhanced green fluorescent protein tag (eGFP_4_) to the *Ztgcs1* gene in the wildtype strain IPO323 (Kema and Van Silfhout, 1997). Using a fusion of multiple eGFP significantly increases the fluorescent signal intensity, allowing visualization of individual cell wall synthases in the living cell (Schuster et al., 2016). We cultured the resulting strain strains IPO323_G_3_Chs5 and IPO323_G_4_Gcs1 in potato dextrose media (PDB) at 25 °C, which induces hyphal growth, and observed eGFP_3_-ZtChs5 and eGFP_4_-ZtGcs1, using laser-based epi-fluorescent microscopy. We found that both fluorescent fusion proteins showed continuous directed motility, indicated by a diagonal line in a kymograph (Fig. 1B and C). Motility of both enzymes occurred at ∼2 µm/s (Fig. 1D), but was rare, with only ∼8 signals passing a given point in the hypha per minute (Fig. 1E). This transport frequency is relatively low (e.g. in *N. crassa*, it has been suggested that 38,000 vesicles per minute fuse with the growing hyphal apex; Collinge and Trinci, 1974). However, *N. crassa* hyphae extend ∼12 µm/minute (Lopez-Franco et al., 1994), whereas *Z. tritici* hyphae grow 115-times slower (0.104 ± 0.051 µm/minute, n = 15), which may explain the low vesicle transport frequency. Alternatively, chitin synthase-containing chitosomes may represent a small sub-population of all secretory vesicles in the hypha.

In hyphae of *U. maydis*, long-range delivery of class V chitin synthase depends on microtubules (MTs, Treitschke et al., 2010). We tested for such a role of MTs in *Z. tritici* by disrupting these cytoskeletal fibers with the inhibitor benomyl, which effectively disassembles the tubulin polymers in *Z. tritici* (Schuster et al., 2015). Treatment of 300 µM benomyl abolished the long-range motility of eGFP_3_-ZtChs5 and eGFP_4_-ZtGcs1 (Fig. 1F). We conclude that delivery of class V chitin synthase in hyphae of *Z. tritici* most depends on microtubules and associated motor proteins. We also applied Latrunculin A, an inhibitor that disrupts F-actin in *Z. tritici* (Kilaru et al., 2017). This treatment did not abolish motility, but lowered slightly the transport frequency (Fig. 1G). These results confirm findings in *Aspergillus nidulans* (Penalva et al., 2017), another member of the Pezizomycotina, and in the basidiomycete *U. maydis* (Schuster et al., 2012, Treitschke et al., 2010), showing that both cytoskeletal elements are involved in tipwards motility of secretory vesicles. In the ascomycete *N. crassa*, delivery of the chitin synthase CHS-1 appears to depend on F-actin, rather than MTs (Sanchez-Leon et al., 2011). However, it is not clear if all chitin synthase travel in the same vesicle. Indeed, fluorescent CHS-3 and CHS-6, as well as CHS-1 show different localizations in the living hypha of *N. crassa* (Riquelme et al., 2007, Sanchez-Leon et al., 2011). Thus, it is possible that different sub-populations of chitosomes utilize different cytoskeletal transport systems. Further research is required to elucidate this notion.

**Fig. 2:**
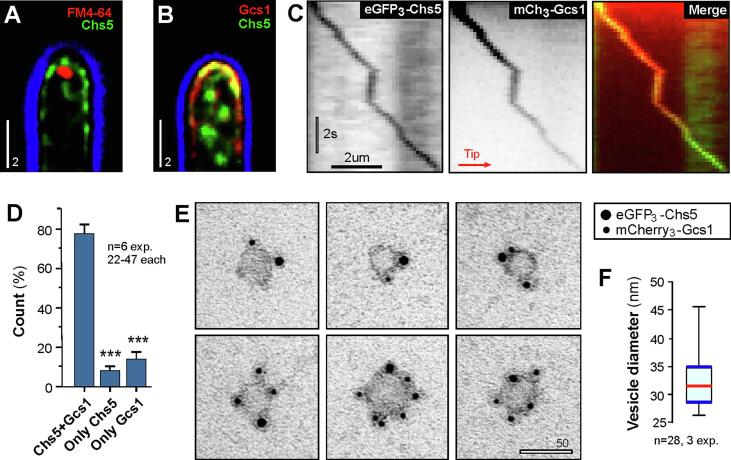
Co-visualization of fluorescent chitin and glucan synthase in hyphae of *Z. tritici.* (A) Co-visualization of fluorescent Chs5 (green) and the SPK, labelled with the dye FM4-64 (red) in a hypha of *Z. tritici*. Note that the fluorescent enzymes is not located in the SPK, which contradicts findings in *N. crassa* (Fajardo-Somera et al., 2015, Verdin et al., 2009). The edge of the cell is indicated in blue. Sale bar: 2 µm. (B) Co-visualization of eGFP_3_-ZtChs5 (green) and mCherry_3_-ZtGcs1 (red) at the tip of a *Z. tritici* hypha. Both cell wall synthases largely co-localize in the apical plasma membrane, resulting in a yellow signal. No apical accumulation of mCherry_3_-ZtGcs1 is visible, suggesting that the putative β(1,3)-glucan synthase is not concentrating in the SPK. Again, this localization differs from reports in *N. crassa* (Sanchez-Leon et al., 2015a, Sanchez-Leon et al., 2015b). The edge of the cell is indicated in blue. Scale bar: 2 µm. (C) Kymographs showing directed co-motility of eGFP_3_-ZtChs5 and mCherry_3_-ZtGcs1 in *Z. tritici*. The direction relative to the hyphal tip is indicated by an arrow. Note that both signals move at the same speed and pause at the same time, suggesting that they are located to the same vesicle. Horizontal bar: 2 µm, vertical bar: 2 s. (D) Bar chart showing the spatial relationship of eGFP_3_-ZtChs5 and mCherry_3_-ZtGcs1 during directed motility in living cells. Most signals co-localize during motility in the cell. Bars represent mean ± SEM, sample size *n* is indicated. Statistical testing used a Student’s *t*-test; Triple asterisk indicate statistical difference to co-localization of both enzymes (Chs5 + Gcs1) at *P* < 0.0001. (E) Immuno-gold–labelling experiments, showing localization of eGFP_3_-ZtChs5 (large nano-gold particles) and mCherry_3_-ZtGcs1 (small nano-gold particles) on vesicles membranes. Both cell wall synthases localize to the same vesicles in various numbers. Note that signals are spatially separated, suggesting that the enzymes do not form super-complexes. Bar represents 50 nm. (F) Graph showing the diameter of cell wall synthase-carrying vesicles, estimated from immuno-gold preparations. Data given as a Whiskers' plot, with 25/75 percentiles indicated as blue lines, median as a red line, and minimum and maximum values as Whiskers ends; the sample sizes *n* is 28 from 3 preparations. (For interpretation of the references to color in this figure legend, the reader is referred to the web version of this article.)

In *N. crassa*, fluorescent β(1,3)-glucan synthase and chitin synthase travel in different sub-populations of vesicles (Riquelme et al., 2007, Sanchez-Leon et al., 2015a, Sanchez-Leon et al., 2015b, Sanchez-Leon et al., 2011, Verdin et al., 2009). Consequently, both vesicles concentrate in different regions of the Spitzenkörper, with numerous chitin synthases (CHS-1, CHS-2, CHS-3, CHS-5, CHS-7) being located in microvesicles that comprise the core of this organelle (Fajardo-Somera et al., 2015), while β(1,3)-glucan synthase was found in macrovesicles that form the periphery of the SPK (Sanchez-Leon et al., 2015a, Sanchez-Leon et al., 2015b, Verdin et al., 2009). We set out to investigate if the chitin synthase ZtChs5 locate to the SPK in *Z. tritici*. To this end, we visualized the SPK in strains expressing eGFP_3_-ZtChs5, using FM4-64, which stains the SPK in filamentous fungi (Fischer-Parton et al., 2000, Harris et al., 2005, Hoffmann and Mendgen, 1998). Co-visualization of eGFP_3_-ZtChs5 and FM4-64 revealed that the chitin synthase does not accumulate in the SPK. Instead, the enzymes locates to the apical plasma membrane (Fig. 2A). This localization in the apical plasma membrane was also reported for class I chitin synthase in *A. nidulans* (Hernández-González et al., 2018), but was not found in *N. crassa* (Fajardo-Somera et al., 2015). However in both these fungi, chitin synthases concentrate in the SPK (Fajardo-Somera et al., 2015, Riquelme et al., 2007, Hernández-González et al., 2018). Why chitin synthases do not concentrate in the Spitzenkörper in *Z. tritici* is not known, but may be linked to the more rapid growth rates of *N. crassa* hyphae (Lopez-Franco et al., 1994) and *A. nidulans* hyphae (∼1.8 µm/minute; Takeshita et al., 2017). We thus speculate that rapid expansion of the hyphal apex in *N. crassa* and *A. nidulans* supports the establishment of the Spitzenkörper as a “vesicle supply center” from where chitin synthase-containing secretory vesicles are released to fuse with the plasma membrane (Bartnicki-Garcia et al. 1989). More slowly growing hyphae, such as in *Z. tritici*, may not require such an intermediate “distribution depot” and here vesicles fuse directly with the hyphal tip.

We next investigated if eGFP_3_-ZtChs5 and mCherry_3_-ZtGcs1 co-localize at the plasma membrane. Simultaneous visualization of both enzymes in growing hyphae of strain IPO323_G_3_Chs5_C_3_Gcs1 demonstrated that both cell wall synthases co-localize at the apical plasma membrane (Fig. 2B). mCherry_3_-ZtGcs1 did not accumulate in the SPK, too, confirming the difference to *N. crassa.* We next set out to investigate eGFP_3_-ZtChs5 and mCherry_3_-ZtGcs1 during motility to the hyphal tip. We found that eGFP_3_-ZtChs5 and mCherry_3_-ZtGcs1 co-localize during intracellular transport (Fig. 2C; Video S1) in almost 80% of all observed vesicles (Fig. 2D). This results is in good agreement with *U. maydis* cells (∼75% co-localization; Schuster et al., 2016). In *U. maydis*, this result was further supported by immuno-gold detection of fluorescent proteins on purified vesicle membranes (Schuster et al., 2016). We followed this strategy, using strain IPO323_G_3_Chs5_C_3_Gcs1, and confirmed the presence of eGFP_3_-ZtChs5 and mCherry_3_-ZtGcs1 on small vesicles (Fig. 2E). These vesicles had a diameter of ∼32 nm (Fig. 2F). While this dimension is slightly smaller than the size of chitosomes (40–70 nm in *Mucor rouxii*; Bracker et al., 1976), it corresponds well with the size of synaptic vesicles (Riveros et al., 1986).

Video S1
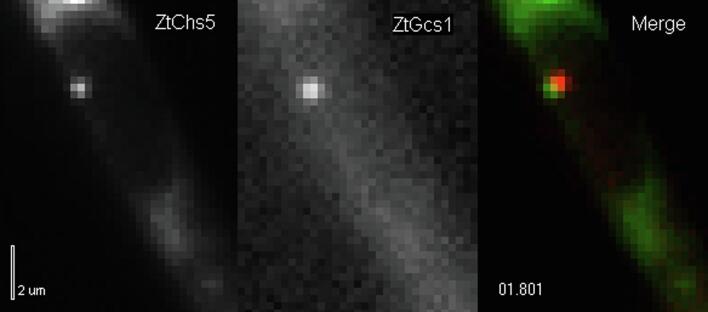

In this Video Article, we investigated the localization and motility of fluorescent class V chitin synthase and β(1,3)-glucan synthase in the ascomycete *Z. tritici*. This fungus is closely-related to *N. crassa*, with both belonging to the Pezizomycotina. Despite this evolutionary similarity, our work reveals some fundamental differences between *Z. tritici* and *N. crassa*: (1) Whilst chitin synthases and glucan synthases in *N. crassa* localize to the SPK, but not the plasma membrane (Fajardo-Somera et al., 2015, Sanchez-Leon et al., 2015a, Sanchez-Leon et al., 2015b), they locate to the plasma membrane but not the SPK in *Z. tritici*; (2) In *Z. tritici*, chitin synthase and glucan synthase travel in the same small vesicle, whereas both enzymes locate in different vesicle populations in *N. crassa* (Riquelme et al., 2007, Sanchez-Leon et al., 2015a, Sanchez-Leon et al., 2015b, Sanchez-Leon et al., 2011, Verdin et al., 2009); (3) Finally, while delivery of chitin synthase to the hyphal tip in *N. crassa* hyphae depends only on F-actin (Sanchez-Leon et al., 2011), it is largely based on MTs in *Z. tritici.* Surprisingly, in all these points, *Z. tritici* resembles findings in the basidiomycete *U. maydis* (Schuster et al., 2016, Schuster et al., 2012, Treitschke et al., 2010). Thus, the important conclusion of this study is that the co-delivery of cell wall synthases in one vesicle, and their transport along MTs, is not restricted to basidiomycetes, but could be of greater importance for hyphal growth in filamentous fungi than previously anticipated. At present, we can only speculate on why *N. crassa* has established a fundamentally different mechanism of delivery of cell wall synthases. One possible explanation is that the rapid growth rate of *A. nidulans* and *N. crassa* is supported by delivery and fusion of a large number of vesicles with the hyphal tip. Achieving this may require a different secretion system, in which the Spitzenkörper is a “vesicle supply center” (Bartnicki-Garcia et al., 1989) for co-ordinated exocytosis of cell wall synthases in different sub-populations of vesicles. In slow-growing hyphae, such as *Z. tritici*, vesicle fusion rates are much lower, which may be supported by Spitzenkörper-independent random exocytosis and which would require co-delivery of cell wall synthases in the same secretory vesicle. Future studies will address this interesting notion.

## 3 Methods

### 3.1 Bioinformatics

The class V chitin synthase ZtChs5 and the putative β(1,3)-glucan synthase ZtGcs1 were identified by screening public databases using BLASTp (https://blast.ncbi.nlm.nih.gov/Blast.cgi) with published homologues from *U. maydis* (see Figure legend 1 for Genbank accession numbers). Domain predictions were with InterPro (http://www.ebi.ac.uk/interpro/) and sequence identity/similarity using EMBOSS Needle (https://www.ebi.ac.uk/Tools/psa/emboss_needle/).

### 3.2 Molecular cloning

All vectors generated by yeast *in vivo* recombination (Raymond et al., 1999), following published procedures (Kilaru and Steinberg, 2015).

*p-3eGFP-ZtChs5*. A 1474 bp fragment of *Ztchs5* promoter (a silent point mutation was introduced to remove the *Bsr*GI restriction site), hygromycin resistance cassette, a 717 bp fragment from eGFP ORF and 964 bp of *ZtChs5* were recombined in *S. cerevisiae* (Kilaru and Steinberg, 2015). This vector was linearized using *Bsr*GI and two additional copies of eGFP introduced giving plasmid p-3eGFP-ZtChs5.

*p-4eGFP-Gcs1*. This plasmid was generated by fusing 830 bp from *Ztgcs1* 5′ upstream region, hygromycin resistance cassette, 930 bp of *Ztgcs1* promoter, a 723 bp fragment from mCherry ORF and the first 730 bp of the *Ztgcs1* gene using yeast *in vivo* recombination. This vector was linearized using *Bsr*GI and three additional copies of eGFP introduced, giving plasmid *p4eGFP-Gcs1*.

*p3mCherry-Gcs1*. A 830 bp fragment from Gcs1 5′ upstream region, G418 resistance cassette, 930 bp of *gcs1* promoter, a 723 bp fragment from mCherry ORF and the first 730 bp of Gcs1 ORF, recombined in *S. cerevisiae*. This vector was linearized using *Bsr*GI and two additional copies of mCherry introduced giving plasmid *p3mCherry-Gcs1*. Vectors were transformed into *A. tumefaciens* and *A. tumefaciens*-mediated transformation of *Z. tritici* performed as described (Kilaru et al., 2015, Kilaru et al., 2017). Correct integration of vectors was confirmed by Southern blot hybridization, following standard procedures.

### 3.3 Fungal growth conditions

All *Z. tritici* strains were stored at −80 °C as glycerol stocks (Cells were maintained as glycerol stocks (NSY glycerol; nutrient broth, 8 g/l; yeast extract, 1 g/l; sucrose, 5 g/l; glycerol, 700 ml/l, Sigma Aldrich, UK). For use in experiments, aliquots were placed on YPD agar plates YPD agar (yeast extract, 10 g/l; peptone, 20 g/l; glucose, 20 g/l; agar, 20 g/l, Sigma Aldrich, UK) and grown at 18 °C for 5 days. Hyphal growth was induced by transferring cells to liquid PDB medium (24 g/l potato dextrose broth, Sigma Aldrich, UK) and additional growth for 24 h at 25 °C, shaking at 150/200 rpm.

### 3.4 Live cell imaging of secretory cell wall synthases

Fluorescence microscopy was performed as described (Schuster et al. 2016). To visualize and quantify motility of eGFP_3_-ZtChs5 and eGFP_4_-ZtGcs1 hyphae were photo-bleached 5–10 µm behind the tip, followed by acquisition of 100 plains. Flux and velocity was analyzed in kymographs generated in MetaMorph 7.8.9.0 (Molecular Devices, Wokingham, UK). To test the importance of the cytoskeleton cells were treated with latrunculin A (Molecular Probes/Invitrogen, Paisley, UK) or benomyl (Sigma–Aldrich Chemie GmbH, Munich, Germany) as described (Kilaru et al., 2015). Co-visualization of eGFP_3_-ZtChs5 and mCherry_3_-ZtGcs1 was done after photo-bleaching of hyphal cells followed by simultaneous acquisition of red- and green fluorescence, using the Dual-View Microimager (Photometrics, Tucson, USA). Kymographs of both channels were generated and overlaid in MetaMorph and co-motility analyzed. The Spitzenkörper was stained with the dye FM4-64 (Molecular Probes/Invitrogen, Paisley, UK), as described in Guo et al. (2015).

### 3.5 Determining growth rate and hyphal diameter

To determine the hyphal growth rate of *Z. tritici*, cells were placed on agar pads and a bright-field image was taken. After 5–10 min, a second image was acquired and merged with the initial image. Hyphal extension over time was measured in these merged images, using the software MetaMorph.

### 3.6 Ultrastructural studies of purified secretory vesicles

Secretory vesicles were purified and visualized as described (Schuster et al., 2016). GFP and mCherry was detected using rabbit anti-GFP (ab6556, Abcam, UK) and rabbit anti-mCherry antibody (antibody PM005, MBL International, USA), TEM was done as described in (Schuster et al., 2016).

### 3.7 Statistical analysis

Statistical analysis was done using GraphPad Prism 5 (GraphPad, San Diego, USA). Firstly, data sets were tested for normal distribution using a Shapiro-Wilk test. Non-normal distributed data sets (P ≤ 0.05) were compared using a nonparametric Mann-Whitney test.
